# Scrub Typhus: Report of Three Cases From Rural Nepal and a Brief Literature Review

**DOI:** 10.7759/cureus.18376

**Published:** 2021-09-29

**Authors:** Jeevan Gautam, Randhir S Yadav, Shumneva Shrestha, Bishnu Mohan Singh, Renusha Maharjan

**Affiliations:** 1 Internal Medicine, California Institute of Behavioral Neurosciences and Psychology, Fairfield, USA; 2 Department of Pediatrics, Tribhuvan University Institute of Medicine, Kathmandu, NPL; 3 Division of Clinical and Translational Research, Larkin Community Hospital, Miami, USA; 4 Internal Medicine, Hetauda City Hospital, Hetauda, NPL; 5 Department of Surgical Nursing, Institute of Medcine, Kathmandu, NPL

**Keywords:** scrub typhus, case report, empirical treatment, eschar, rural nepal

## Abstract

Scrub typhus is endemic among farmers in the rural southern part of Nepal. It is grossly underdiagnosed due to a lack of clinical suspicion and inadequate testing facilities. The most common clinical features of the disease include fever, rashes, vomiting, myalgia, and eschar. The disease may present with ocular changes such as conjunctival injection, gastrointestinal features such as hepatitis and splenomegaly, acute kidney injury (AKI), or neurological findings in the form of meningoencephalitis. Herein, we present a report of three cases of scrub typhus from a rural part of South-west Nepal who failed to receive appropriate treatment initially. One of the patients recovered well with the treatment, the other developed AKI but recovered over the next few weeks. One of the patients died due to sepsis/multiorgan failure secondary to scrub typhus. While managing such cases in places with limited diagnostic facilities, the incorporation of early appropriate empirical therapy for scrub typhus after a careful clinical assessment prevents complications and saves lives.

## Introduction

Scrub typhus is a vector-borne disease caused by *Orientia tsutsugamushi*, a Gram-negative intracellular bacterium, and transmitted by the larva of trombiculid mite. It is an important cause of acute febrile illness in Southeast Asia and Pacific islands [1] including the Southern belt of Nepal where it emerges as an epidemic during the monsoon and autumn. It is highly prevalent in rural areas where it is difficult to timely diagnose and treat the disease [2]. In Nepal, the incidence of scrub typhus is on rising, for instance, there were 101 cases reported in 2015, 831 cases in 2016, and 1271 cases in 2019 [3,4]. The disease is associated with occupational or agricultural exposures including exposure to piles of wood, bushes, domestic animals, rodents, and active rice fields [5]. Most of the cases present with fever, myalgia, rash, headache, abdominal pain, and sometimes, signs of organ damage [6]. Due to a lack of clinical suspicion and lab diagnosis, fatal complications like acute respiratory distress syndrome (ARDS), meningoencephalitis, pneumonitis, acute kidney injury (AKI), and myocarditis often occur in patients who are not treated early [6]. We are reporting three cases of scrub typhus from the rural part of Nepal with a brief discussion on the relevant literature.

## Case presentation

All three cases presented to the outpatient clinic of a rural primary health center (PHC) with a chief complaint of fever. Our PHC is a state-owned rural health facility providing basic diagnostic and curative services including essential drugs and laboratory facilities. The relevant details of the three cases are summarized in Table 1.

**Table 1:** Summary of cases AE: acute exacerbation, COPD: chronic obstructive pulmonary disease, ESR: erythrocyte sedimentation rate, CRP: C-reactive protein, BUN: blood urea nitrogen, ELISA: enzyme-linked immunosorbent assay, AKI: acute kidney injury, NA: not available.

Details			Case 1	Case 2	Case 3
Age			45 years	72 years	27 years
Gender			Male	Female	Female
Occupation			Farmer/teacher	Farmer	Farmer/student
Chief complaint			Intermittent fever for 8 days, profuse sweating, headache, myalgias	High-grade fever for 6 days, fast breathing, dry cough, decreased alertness	Fever for 14 days, headache, abdominal pain, vomiting, myalgia, irritability, irrelevant talk
Treatment received earlier			Oral acetaminophen and parenteral ceftriaxone	Oral levofloxacin and parenteral ceftriaxone	Oral acetaminophen and parenteral ceftriaxone
Comorbidity			None	COPD	None
Vitals	SpO_2_		92%	90%	>90%
	Blood pressure		110/60 mmHg	90/50 mmHg	100/60 mmHg
	Pulse		116/min	110/min	112/min
	Respiratory rate		18/m	28/m	20/m
	Temperature		103 °F	102 °F	104 °F
General examination			Suffused conjunctiva, mild dehydration	Dehydrated, somnolent	Disoriented to time and place, irritable
Inspection for Eschar			1.8 cm × 1 cm eschar on the right infra axillary region of the chest	2 cm × 1 cm eschar on the right loin	Healed eschar
Systemic examination			Normal	Scattered wheezes over bilateral lungs	Normal
Relevant lab findings		Leukocyte count	13000/c.mm	16,000/c.mm	NA
		Liver function tests	Mildly elevated liver enzymes	NA	NA
		ESR	Raised	NA	NA
		CRP	Raised	NA	NA
		Chest X-ray	NA	COPD findings with no active changes	NA
		BUN	36 mg/dl	NA	NA
		Serum creatinine	1.7 mg/dL	NA	NA
		ELISA for scrub typhus	Positive	Positive	Positive (confirmed via phone from the other center where the patient was referred)
Treatment			Supportive treatment empirical doxycycline 100 mg PO bd for 7 days (at the PHC)	Supportive treatment standard treatment for COPD empirical doxycycline 100 mg PO bd for 7 days (at the PHC)	Referred to tertiary center after primary management and the first dose of oral doxycycline 100 mg
Diagnosis			Scrub typhus with early-stage AKI	Scrub typhus with AE of COPD	Scrub typhus (with sepsis/suspected meningoencephalitis)
Outcome			Recovered with no adverse or unanticipated outcomes. Creatinine level normalized over the next 2 weeks.	Recovered with no adverse or unanticipated outcomes	Expired

**Figure 1:**
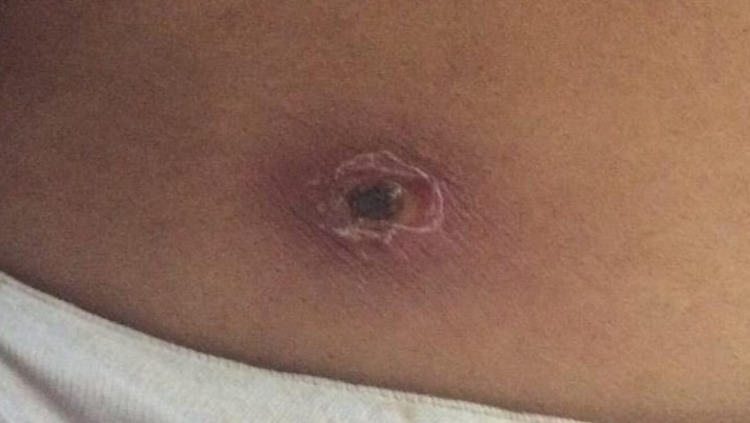
An 1.8 cm X 1.1 cm eschar in infra axillary region of the right chest

Figure 1 shows 1.8 cm × 1 cm eschar noted on the right infra axillary region of the chest of the first patient (Case 1).

**Figure 2:**
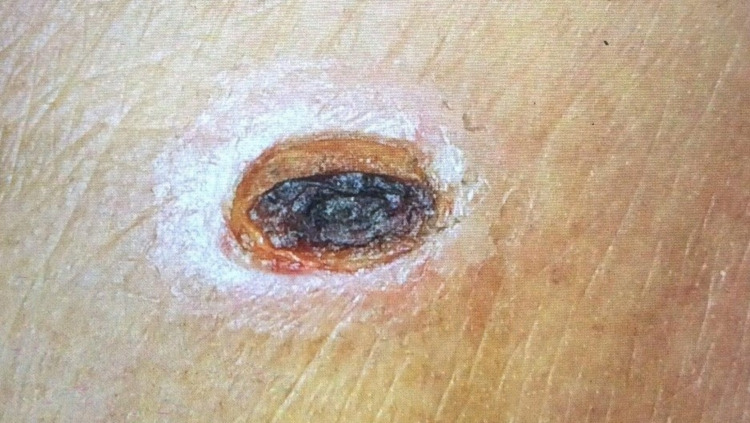
An 2 cm × 1.2 cm eschar on the right loin

Figure 2 shows 2 cm × 1 cm eschar noted on the right loin of the second patient (Case 2).

None of the three patients had received appropriate antibiotics prior to their presentation to our center. We were able to successfully diagnose based on clinical evaluation and treat two patients (cases 1 and 2) as we could timely intervene. In both cases, rapid diagnostic tests (RDT) were negative for the malarial parasite. Serology of Leptospira, dengue, human Immunodeficiency virus (HIV), hepatitis B surface antigen (HBsAg) and hepatitis C virus, and Widal test for *Salmonella typhi* and *S. paratyphi* were also negative. They recovered well (analyzed on the basis of the resolution of symptoms) before the reports were obtained positive for scrub typhus on the eighth and seventh day, respectively. Case 1 had his renal function tests normalized over the next two weeks. Case 3 presented very late, on the fourteenth day of onset of illness. We referred her to a higher center. We followed up on her status later via phone call. It was reported that she had developed increased confusion and signs of septic shock including oliguria and hypotension. Her test results revealed leukocytosis, thrombocytopenia, and deranged liver functions but were negative for the malarial parasite, Leptospira, dengue, HIV, HBsAg, HCV, *S. typhi*, and *S. paratyphi* and she expired on the second day of admission. An autopsy was not performed but the cause of death was presumed to be sepsis causing multi-organ failure including meningoencephalitis which proved to be secondary to scrub typhus as her blood sample came positive for scrub typhus later.

## Discussion

Scrub typhus is emerging as a potential health concern in Nepal due to poor health care, limited diagnostic facility, and the epidemiological trend of the disease [2]. The common clinical features are fever with chills, rashes, and non-specific symptoms like headache, myalgia, sweating, and vomiting [7]. Patients may manifest gastrointestinal (hepatitis, splenomegaly), respiratory (pneumonia, ARDS), neurological (meningoencephalitis), or ocular (conjunctival injection, subconjunctival hemorrhage) symptoms [6]. The clinical features may also resemble other tropical infections like enteric fever, malaria, dengue, or leptospirosis [6]. But eschar is a characteristic finding in about half the patients with proven scrub typhus [6]. Previous literature has reported the presence of eschar in 43.5-87% of the cases from different studies [7-9]. It is pathognomonic of scrub typhus [10] but is often unreported by patients as it is painless and non-pruritic [11]. A meticulous physical examination to search for eschar often helps to make a diagnosis of scrub typhus, so that treatment can be initiated early.

Delayed diagnosis and treatment results in severe complications like ARDS, septic shock, and multisystem organ failure causing death [6]. Renal involvement including AKI is a common systemic complication of scrub typhus. In a study, among 502 patients of scrub typhus diagnosed by ELISA, the majority had stage 1 AKI [12] which mostly developed on the fifth to the sixth day. Further, 18.73% and 3.94% of them required intensive care unit (ICU) admission and hemodialysis, respectively [12]. In our case 1, the patient, fortunately, presented during the early stage of AKI and recovered well. Delay in treatment while waiting for the confirmation of the disease would have worsened the prognosis. It can be difficult to treat the disease during the late stage even with appropriate antibiotics because of complications. As in our third case, mortality is usually because of late presentation and delay in diagnosis due to the lack of laboratory methods in many geographical areas [10]. In a study in India, out of 116 scrub typhus patients admitted to the medical critical care unit, 87.9% required ventilatory support and hospital mortality was 24% [8]. In a large cohort study of 623 patients hospitalized with scrub typhus, the mortality rate was 9%. Shock requiring vasoactive agents, central nervous system (CNS) dysfunction, and renal failure were found to be the independent predictors of mortality [7]. Similarly, mortality in ICU admitted patients of scrub typhus was found to be 20% in a recent study in Nepal [13]. Even with the laboratory tests, 94.3% of the cases were not definitively diagnosed, according to a surveillance data [1]. Weil-Felix test results are usually negative during the early stages of the disease [14] and only 50% of patients have positive tests during the second week of illness [1]. Limited access to rapid tests and low sensitivity of the tests, especially early in the disease course highlights the importance of early clinical diagnosis of this easily curable illness.

In 2015, a mortality due to a presumptive diagnosis of bilateral pneumonia with sepsis/leptospirosis was reported in India, which was later proved to be scrub typhus [15]. In an outbreak of scrub typhus in North India in 2009, due to a delay in the lab report, all the patients were empirically treated with doxycycline with a 100% positive outcome [16]. Rapid resolution after taking antibiotics is so typical that it can be used as a diagnostic test for *Rickettsia tsutsugamushi* [10]. Such instances are excellent lessons for the physicians to treat the condition early enough to lessen hospital stay and avoid mortality. Morbidity and mortality associated with scrub typhus can be reduced significantly by early recommended empiric therapy in clinically suspected cases of scrub typhus [13].

Doxycycline is the drug of choice, but it is inconsistently used in Nepal. On a Cochrane review in 2018, the use of tetracycline, doxycycline, azithromycin, and rifampicin were found effective treatment options with little or no difference among them and with few treatment failures [17]. Also, Basnyat has given a plausible rationale on the empirical use of doxycycline over rifampicin in Nepal where both scrub typhus and tuberculosis are common [18]. Azithromycin is an appropriate alternative for children under eight years of age and pregnant women [19]. In Patan Hospital, a tertiary center of central Nepal, ceftriaxone and doxycycline are used for undifferentiated febrile illnesses with a temperature above 38 °C for four days, after ruling out malaria and dengue. A similar syndromic approach is practiced in another tertiary hospital in Eastern Nepal [20].

We also need to consider the limitations to the empirical treatment of suspected scrub typhus. The emergence of resistant strains is a serious implication of starting empirical therapy, posing a future threat in treatment for scrub typhus. Due to the variable occurrence of eschar in the patients in previous studies, its absence does not rule out the disease [16]. Also, all eschars cannot be attributed to scrub typhus. It is imperative to differentiate scrub typhus from other diseases such as malaria, arbovirus infections, e.g., dengue, leptospirosis, meningococcal disease, typhoid, infectious mononucleosis, and HIV which may mimic similar clinical features [12]. Co-infection with leptospirosis, dengue, and malaria has also been reported. So, a high degree of vigilance to not overlook an alternative or overlapping possibility cannot be emphasized enough. However, as correct drug therapy shows the rapid response in the cases of scrub typhus [16], it is prudent to incorporate cheap, yet effective, drugs such as doxycycline in the treatment protocol of acute febrile illness in all patients during the epidemic season in Nepal.

## Conclusions

Availability, cost, time, and sensitivity of lab testing of scrub typhus are major problems in the rural areas of Nepal. A presumptive diagnosis of scrub typhus can be made on the basis of clinical features such as fever with chills, headache, vomiting, rashes, and myalgia and fairly specific clinical signs like eschar and epidemiological evaluation. Health care providers should have a high degree of clinical suspicion, conduct a careful physical examination, and remain vigilant for alternate diagnosis in such settings where scrub typhus is common. In suspected cases of scrub typhus, incorporation of empirical treatment with appropriate antibiotics while waiting for confirmation and ruling out other possibilities, would timely prevent future complications, disease burden, and death. Local health care providers need to be adequately trained and efforts should be made on equipping the rural health centers with diagnostic tools.
